# Short-Term Effects of Adding Topical Ketorolac to Intravitreal Bevacizumab in Diabetic Macular Edema: A Crossover Randomized Clinical Trial

**DOI:** 10.18502/jovr.v19i1.15424

**Published:** 2024-03-14

**Authors:** Alireza Ramezani, Hossein Molazem, Morteza Entezari, Homayoun Nikkhah, Saman Rezanejad, Mehdi Yaseri

**Affiliations:** ^1^Ophthalmic Epidemiology Research Center, Research Institute for Ophthalmology and Vision Science, Shahid Beheshti University of Medical Sciences, Tehran, Iran; ^2^Ophthalmic Research Center, Research Institute for Ophthalmology and Vision Science, Shahid Beheshti University of Medical Sciences, Tehran, Iran; ^3^School of Medicine, Shahid Beheshti University of Medical Sciences, Tehran, Iran; ^4^Imam Hossein Medical Center, Shahid Beheshti University of Medical Sciences, Tehran, Iran; ^5^Negah Aref Ophthalmic Research Center, Shahid Beheshti University of Medical Sciences, Tehran, Iran; ^6^Torfeh Medical Center, Shahid Beheshti University of Medical Sciences, Tehran, Iran; ^7^Department of Epidemiology and Biostatistics, Tehran University of Medical Sciences, Tehran, Iran

**Keywords:** Bevacizumab, Diabetic Macular Edema, Intravitreal, Topical Ketorolac

## Abstract

**Purpose:**

To evaluate the short-term additive effects of topical ketorolac to intravitreal bevacizumab (IVB) in the management of center-involved diabetic macular edema (CI-DME).

**Methods:**

In a randomized double-masked placebo-controlled crossover clinical trial, eyes with CI-DME and the best-corrected visual acuity (BCVA) between (20/40) and (20/400) were included. These eyes should have had at least one intravitreal anti-VEGF injection in the preceding two months. They were randomized into two groups; while both groups received two IVB injections with a six-week interval, one group received topical ketorolac every 6 hr in the first interval and artificial tears every 6 hr as a placebo in the second interval and the other group received the same medications using a crossover method. The main outcome measures were changes in BCVA and central macular thickness (CMT).

**Results:**

Fifty-seven eyes of 35 patients with CI-DME were included in the study. The mean BCVA improvement was –0.09 ± 0.47 logMAR in the periods of receiving ketorolac and –0.03 ± 0.12 logMAR in the periods of placebo treatment, respectively (*P* = 0.99). Corresponding changes in CMT were –13.1 ± 170.1 and +11.7 ± 157.7 µm in the ketorolac and placebo periods, respectively (*P* = 0.322). The treatment effect was not statistically significant regarding both BCVA and CMT changes. Statistical analysis also disclosed that the carryover effect was insignificant for BCVA and CMT. Although the period effect was not significant for BCVA, it was at a meaningful level for CMT changes (*P* = 0.012).

**Conclusion:**

This crossover clinical trial demonstrated that in the course of DME treatment with IVB injections, topical ketorolac did not have any additive beneficial effect at least during a six-week period.

##  INTRODUCTION

Diabetic macular edema (DME) is one of the major reasons for visual impairment in the working-age population and the leading cause of reduced visual acuity in patients with diabetes mellitus.^[1,2]^ It occurs in nearly 12% of patients with diabetic retinopathy.^[3,4]^

For several decades, laser photocoagulation was the standard treatment for patients with clinically significant DME; however, its beneficial effect was only an approximate 50% reduction in the rate of moderate vision loss at three years after treatment.^[5]^ In addition, use of the laser would leave macular scars that increase in size over time and can cause secondary vision loss.^[6]^

Nowadays, anti-vascular endothelial growth factor (anti-VEGF) therapy has become the first-line treatment for DME. Currently available intravitreal anti-VEGF agents include bevacizumab, ranibizumab, and aflibercept. Different trials have proven their beneficial effect in DME treatment; however, suboptimal responders and burden of frequent injections have stimulated the development of novel approaches.^[7]^

Multiple types of non-steroidal anti-inflammatory drugs (NSAIDs) with various routes of administration have also been described for the treatment of DME. Intravitreal injections of these drugs have been investigated for the treatment of DME in many studies.^[8,9,10,11,12,13,14]^ Since topical forms can penetrate into the vitreous cavity and lower the vitreal prostaglandin E2 concentration,^[15,16]^ they have also been used for various causes of macular edema including DME.^[17,18,19,20,21]^ To the best of our knowledge, however, the literature lacks high-quality evidence for using topical NSAIDs in DME treatment and a recently published review article did not identify any randomized

controlled trials regarding this issue.^[22]^ Therefore, we performed this study to investigate whether addition of topical NSAID eye drops to the routine DME treatment of intravitreal injections of anti-VEGF could be beneficial or not. This trial was conducted as a crossover study to eliminate any possible confounding factors during the study.

##  METHODS

This randomized double-masked placebo-controlled crossover clinical trial adhered to the tenets of the Declaration of Helsinki and was approved by the Ethics Committee of the Ophthalmic Research Center affiliated to Shahid Beheshti University of Medical Sciences, Tehran, Iran (IR.SBMU.ORC.REC.1398.015). The study protocol was explained to all patients before recruitment and informed consent was obtained from all participants. This study was registered at Clinical-Trials.gov (NCT04119921). The study was performed in two university-affiliated medical centers named Imam Hossein and Torfeh hospitals located in Tehran, Iran.

Of diabetic patients older than 18 years of age, those with center-involving DME with a mean central subfield thickness ≥ 300 micron on optical coherence tomography (OCT) who needed at least two anti-VEGF intravitreal injections in the subsequent 12 weeks were included. A history of intravitreal injections of an anti-VEGF drug within the prior two months was mandatory for enrollment. Media clarity, pupillary dilation, and subject's cooperation sufficient for study protocols were necessary.

We excluded eyes with best-corrected visual acuity (BCVA) better than 20/40 or worse than (20/400) Snellen equivalent, recent ( < 6 months) laser treatment, high-risk or active proliferative diabetic retinopathy (PDR), history of intravitreal or peribulbar

This is an open access article distributed under the Creative Commons Attribution License, which permits unrestricted use, distribution, and reproduction in any medium, provided the original work is properly cited.

corticosteroid injection in the prior three months, history of intraocular surgery (except cataract extraction more than six months before inclusion), history of complicated cataract surgery, and any other ocular diseases including cataract that would affect BCVA. Exclusion criteria also consisted of uncontrolled diabetes (HgbA1C > 8) and one-eyed patients.

All patients initially underwent a thorough ophthalmic examination including BCVA measurement, slit lamp biomicroscopy, measurement of intraocular pressure, fundoscopy, and OCT. Eligible eyes were randomly assigned to two groups. Eyes in both groups were scheduled to receive two 1.25 mg (0.05 ml) intravitreal bevacizumab (IVB) injections (Avastin; Genentech Inc., South San Francisco, CA) with a six-week interval. Additionally, group 1 received topical ketorolac eye drops (Sinarolac; Sina Darou Laboratories Company, Tehran, Iran) every 6 hr in the first interval followed by artificial tear eye drops (Tearlose; Sina Darou Laboratories Company, Tehran, Iran) every 6 hr as a placebo in the second interval. Group 2 received the same eye drops according to a crossover order in the same six-week intervals. The patients were given new eye drop bottles after six weeks, at the time of the second IVB injections. In bilateral cases, each eye was randomized into a different group. Each 100-ml topical ketorolac eye drop contains 0.5 gr ketorolac tromethamine and benzalkonium chloride as a preservative and each 100 ml artificial tear eye drop contains 0.3 gr hydroxypropyl methylcellulose and 0.1 gr dextran 70 and benzalkonium chloride as a preservative.

The BCVA was measured using the Snellen chart and recorded in a logarithm of the minimum angle of resolution (logMAR) scale. We used commercially available equipment Spectralis ${\;}^{TM}$ OCT technology (Heidelberg Engineering, Heidelberg, Germany) to perform OCT mapping. Retinal thickness was measured in a 3-mm diameter circle centered on the fixation point. The mean thickness on the central 1-mm circle was considered as central macular thickness (CMT).

Intravitreal injections were performed under sterile conditions and topical anesthesia with a 30-gauge needle. In bilateral cases, injection of the second eye was performed after two days. Staff members other than study investigators performed the injections to keep the investigators masked.

Randomization was performed by a random block permutation method according to a computer-generated randomization list. A biostatistician performed the random allocation sequencing. The bottles of ketorolac and artificial tear eye drops were similar regarding size and shape and were labeled with unidentified numbers. Therefore, participants and the investigators evaluating the outcome measures including BCVA and CMT measurements were masked to the groups.

Complete ophthalmic examination including BCVA and CMT evaluations were repeated at the termination of each treatment period, that is, at 6 and 12 weeks. The main outcome measure was BCVA changes according to logMAR notation and the second outcome was CMT changes. Any potential complications related to the interventions were recorded.

### Statistical Analysis

Considering a true difference of 0.2 logMAR in BCVA between the treatments, 54 patients were required in this two-treatment crossover study to have a power of 95% to detect the difference at a two-sided 0.05 significance level. This was based on the assumption (obtained from our pilot study with sample size of 6) that the standard deviation of the change in the response variables in two periods is 0.4. In order to consider possible loss of information, we planned to include > 60 subjects into our study.

To describe parameters, mean, standard deviation, median, and range were used. To assess the carryover effect, period effect, and treatment effect simultaneously, we used linear mixed model. In addition, we used another linear mixed model to assess the treatment effect when the data of the two periods are collapsed and also to assess the within-group changes (adjusted for multiple comparison by Sidak method). All statistical analyses were performed using the SPSS software (IBM Corp. Released 2016. IBM SPSS Statistics for Windows, Version 24.0. Armonk, NY: IBM Corp.). *P*-values < 0.05 were considered statistically significant.

##  RESULTS 

Of the 47 patients (81 eyes) who primarily entered the trial, 5 patients who could not attend their prearranged visits and 3 who made mistakes in using the assigned drops were excluded from the final analyses. In addition, cases that needed vitrectomy (four eyes) or peripheral retinal laser photocoagulation (five eyes) during the study period were excluded.

Finally, 57 eyes of 35 patients (28 female and 7 male) with the mean age of 61 ± 7.8 years (range, 41 to 77) were included for statistical analyses. Our entire patient population had type 2 diabetes. History of hypertension, hyperlipidemia, and cigarette smoking were reported in 25%, 25%, and 8.5% of the patients, respectively.

From all eyes, 17.5% were pseudophakic. History of previous macular laser photocoagulation was positive in 12.3% of the eyes. Diabetic retinopathy severity scale was moderate non-proliferative diabetic retinopathy (NPDR), severe NPDR, and regressed PDR in 10.5%, 61.4%, and 28.1% of eyes, respectively. During the preceding two months before the enrolment, 92.9% of the eyes had received one and 7.1% had received two IVB injections.

Table 1 shows the changes of BCVA and CMT in each group and in the two periods. There was no significant difference between the periods of using ketorolac or placebo in both BCVA and CMT changes (*P*-values of the treatment effect = 0.990 and 0.323, respectively). The carryover was also insignificant for both BCVA and CMT changes (*P *= 0.545 and *P *= 0.944, respectively). Although the period effect was not meaningful for BCVA outcome (*P *= 0.565), it reached a statistically significant level for the CMT changes (*P *= 0.012*)*.

In another analysis, we pooled up the data of the two periods of using ketorolac and the two periods of using placebo and compared with each other. The mean BCVA changes were –0.09 ± 0.47 and –0.03 ± 0.12 logMAR and the mean CMT changes were –13.07 ± 170.1 and +11.74 ± 157.67 µm in the periods of using ketorolac and placebo, respectively. The differences between the use of ketorolac and placebo were not significant (*P *= 0.67 for BCVA and *P *= 0.96 for CMT changes).

We also performed subgroup analyses based on the presence of the various possible confounding factors including abnormal lipid profile, hypertension, smoking, and unilaterality of the DME. None of the mentioned factors caused a significant difference in the treatment responses regarding BCVA and CMT changes.

During the study, we did not observe any significant side effect related to the topical eye drops.

**Table 1:** The changes of best-corrected visual acuity and central macular thickness in each group and in the two periods.


		<@orange**Group**		<@orange ***P*** **-values**		
		**0 → ketorolac → placebo**	**0 → placebo → ketorolac**	**Treatment effect**	**Carryover effect**	**Period effect**
BCVA*	Baseline	0.7 ± 0.97	0.38 ± 0.19	0.99	0.545	0.565
	Week 6	0.64 ± 0.79	0.36 ± 0.17		
	Change from baseline	0.07 ± 0.48	0.01 ± 0.15		
	*P*-within	0.914		
	Week 12	0.62 ± 0.89	0.35 ± 0.15		
	Change from baseline	0.08 ± 0.44	0.03 ± 0.16		
	*P*-within			
	Change from baseline	0.01 ± 0.65	0.01 ± 0.09		
	*P*-within			
CMT**	Baseline	485 ± 137	445 ± 106	0.323	0.944	0.012
	Week 6	454 ± 111	425 ± 120		
	Change from baseline	30 ± 82	20 ± 75		
	*P*-within			
	Week 12	457 ± 121	404 ± 115		
	Change from baseline	27 ± 119	40 ± 91		
	*P*-within			
	Change from baseline	-3 ± 101	20 ± 45		
	*P*-within			
	
	
${\;}^{*}$ In logarithm of the minimum angle of resolution (logMAR) scale; ${\;}^{**}$ In microns BCVA, best-corrected visual acuity; CMT, central macular thickness						

##  DISCUSSION

This crossover clinical trial showed that adding topical ketorolac, as an NSAID eye drop, does not have any beneficial effect in eyes under treatment with IVB for CI-DME, in terms of both functional and anatomical outcomes.

Although anti-VEGF therapy is generally considered the first-line therapy for CI-DME, clearly not all DME patients respond favorably to anti-VEGF agents. Even with monthly or near-monthly intravitreal injections of anti-VEGF for the first 12 months of treatment of patients with DME, > 35% fail to achieve ≥ 10-letter improvement in BCVA and > 55% fail to achieve ≥ 15-letter improvement after two years of therapy.^[23]^ In addition, extended follow-up of large trials demonstrated that anti-VEGF injections after the first year could only sustain the observed initial functional and anatomical improvement and did not lead to more visual acuity gain or CMT reduction.^[24,25,26,27,28]^ Therefore, in eyes with such a chronic disease, any intervention beyond frequent anti-VEGF injections that could increase the chance of vision improvement is valuable.

The ophthalmologic utility of NSAIDs is based on the inhibition of COX enzymes and the consequent reduction in circulating prostaglandins that are pro-inflammatory mediators responsible for inducing vasodilation, facilitating leukocyte migration, and blood–ocular barrier disruption. In addition, it is believed that inflammation has a critical role in the development of DME. Therefore, both intravitreal and topical forms of NSAIDs have been tried for treatment of DME. Although intravitreal forms of NSAIDs have shown some promising results in a few studies,^[8,9,10,12,13,14]^ intravitreal anti-VEGF drugs are currently accepted as the first-line treatment of DME. On the other hand,topical forms of NSAIDs would be an attractive alternative treatment option in dealing with such a chronic disease providing an acceptable proven additional benefit to the routine frequent administration of anti-VEGF injections.

Topical NSAIDs are widely used in ophthalmology to decrease inflammation, treat post-cataract surgery cystoid macular edema, reduce pain and photophobia after refractive surgery, and relieve allergic conjunctivitis-related itching.^[29,30,31]^ A number of topical NSAIDs such as bromfenac, diclofenac, flurbiprofen, ketorolac, and nepafenac are formulated for ophthalmic use. They may provide sufficient vitreous concentration to decrease vitreous prostaglandin E2 levels.^[32]^ In the present study, we used topical ketorolac tromethamine. According to some published studies, it can reach to a level in the aqueous and vitreous cavity to lower the prostaglandin E2 concentration, even greater than some other NSAID drops.^[33,34,35,36,37]^

There are many published papers regarding the effects of NSAID drugs in the prophylaxis and treatment of post cataract surgery cystoid macular edema in non-diabetic and diabetic patients. In a review article published in 2015, the authors could not identify any randomized controlled trials for using topical NSAIDs in the treatment of DME.^[22]^ Following that, a pilot study with 17 cases was published and reported the efficacy of topical bromfenac in patients with newly diagnosed DME. The authors reported a significant reduction of CMT, from 465 ± 118 µm at baseline to 388 ± 152 µm post treatment (*P* = 0.02). However, the study was not powered enough to demonstrate a meaningful improvement in BCVA. It should be noted that in their study, the patients did not receive any anti-VEGF injections.^[17]^

In a multicenter, double-masked randomized trial, topical nepafenac 0.1%, three times daily for one year, was used in the treatment of non-center involved DME. They could not find any meaningful effect regarding visual and anatomic outcomes.^[20]^ In another randomized study, nevertheless, the authors found a beneficial effect of three weeks topical ketorolac versus placebo in eyes with focal DME treated by macular photocoagulation. They believed that topical ketorolac might reduce the inflammation caused by photocoagulation and improve the visual outcome.^[21]^ In a case series on 14 eyes, the authors showed a significant decrease in the mean CMT after three months of treatment for DME by administering bromfenac sodium hydrate 0.9 mg/mL eyedrops twice daily; however, the visual outcome was not significantly affected.^[38]^ According to the results of the present study, we could not detect any beneficial effect by adding topical ketorolac to IVB injections in eyes with CI-DME.

Our cases received one injection of IVB as an acceptable, although off-label, drug for the management of DME,^[25,39,40,41,42]^ at the presentation and one injection at the time of crossover. In this way, we simulated a usual treatment strategy that most of our diabetic patients experienced in real life and the only change was the addition of ketorolac eye drop. Additionally, our protocol was based on six-week interval between the injections. It was possible that four-week injections would provide better VA gains as reported in the RISE and RIDE ranibizumab studies.^[26,27,43]^ Nevertheless, this has to be balanced against the long-term visits required in patients with diabetes, who may already be frequent hospital attenders.^[25,44]^

It has been shown in previous studies that most BCVA improvements and CMT reductions occur after the first anti-VEGF injections.^[45,46,47,48,49,50]^ This fact might have presented a sort of bias in a crossover study like ours causing a better response after the first IVB injections as compared to the second period after treatment switching. To reduce this bias, receiving an anti-VEGF injection in the prior two months before enrollment was mandatory in our inclusion criteria making our first injection, the second injection. However, the differences of BCVA and CMT changes between the first and the second periods were not statistically significant.

In the present study, we did not detect a significant improvement in BCVA after two IVB injections. Large trials demonstrated that after primary improvement achieved by initial intensive therapy, BCVA and CMT would only remain stable by retreatment in the following months.^[23][24,25,26,27,28]^ None of our patients were naïve DME cases and had been under anti-VEGF treatment; therefore, not showing a significant improvement after two IVB injections during the study could be expected. Nevertheless, the period effect was significant for CMT reduction during the study period.

Since DME is a chronic disease and the available treatment modalities do not permanently alter its course, we selected a crossover trial design for our study. Through this type of study, we would overcome intra-individual variabilities, various biases, and many confounding systemic factors. These factors are very common in diabetic patients and generally affect the outcome. The principal drawback of the crossover trial is the “carryover” effect and the usual approach to prevent this is to introduce a washout (no treatment) period.^[51]^ However, since the half-life of the NSAIDs has been measured to be very short in the aqueous and vitreous (2.3 hr after intravitreal injection in the rabbit),^[52,53]^ we did not consider a washout period. In addition, the carryover effect in our study was not significant. Another limitation of our study was the possibility of systemic absorption of the topical drops that could affect the result in the contralateral eyes in bilateral cases. In subgroup analysis, however, unilaterality versus bilateral involvement did not demonstrate a significant difference in the results. The same analysis also failed to demonstrate meaningful effect of lipid profile, hypertension, and smoking on the treatment response, emphasizing that the small sample size might affect the results of such analysis.

We performed a post hoc power analysis to determine the power of the study considering the loss of subjects and the maximum observed standard deviation of change (SD = 0.65) in the periods for detection of 0.2 logMAR change in BCVA. It revealed that our study had a power of 62% to detect such differences. In view of this newly observed standard deviation, one needs at least 86, 113, and 140 subjects in a study to have a power of 80%, 90%, and 95%, respectively, to detect a 0.2 logMAR difference in BCVA.

Our study was strengthened due to its double-masked randomized crossover trial design and demonstrated that using topical ketorolac in eyes with CI-DME under treatment with frequent IVB injections provided no additional benefits. However, larger studies with longer follow-up periods and application of other types of topical NSAIDS are warranted.

##  Financial Support and Sponsorship

None.

##  Conflicts of Interest

None.
